# Research on Q-Learning-Based Cooperative Optimization Methodology for Dynamic Task Scheduling and Energy Consumption in Underwater Pan-Tilt Systems

**DOI:** 10.3390/s25154785

**Published:** 2025-08-03

**Authors:** Shan Tao, Lei Yang, Xiaobo Zhang, Shengya Zhao, Kun Liu, Xinran Tian, Hengxin Xu

**Affiliations:** 1 College of Ocean Science and Engineering, Shandong University of Science and Technology, Qingdao 266590, China; 202383190032@sdust.edu.cn (S.T.); zxb@sdust.edu.cn (X.Z.); 202483190077@sdust.edu.cn (X.T.); 2 National Deep Sea Center, Qingdao 266237, China; zsy@ndsc.org.cn (S.Z.); liukun@ndsc.org.cn (K.L.); 3 College of Transportation, Shandong University of Science and Technology, Qingdao 266590, China; xuhx@sdust.edu.cn

**Keywords:** underwater pan-tilt system, energy consumption optimization, automatic wake-up, Q-learning

## Abstract

Given the harsh working conditions of underwater pan-tilt systems, their energy consumption management is particularly crucial. This study proposes an underwater pan-tilt operation method with an automatic wake-up mechanism, which activates only upon target detection, replacing conventional timer-based triggering. Furthermore, departing from fixed-duration observation strategies, we introduce a Q-learning algorithm to optimize operational modes. The algorithm dynamically adjusts working modes based on surrounding biological activity frequency: employing a low-power mode (reduced energy consumption with lower monitoring intensity) during periods of sparse biological presence and switching to a high-performance mode (extended observation duration, higher energy consumption, and enhanced monitoring intensity) during frequent biological activity. Simulation results demonstrate that compared to fixed-duration observation schemes, the proposed optimization strategy achieves a 11.11% improvement in monitoring effectiveness while achieving 16.21% energy savings.

## 1. Introduction

In recent years, the rapid development of marine technology has led to a significant increase in the application of underwater equipment for marine development and exploration [1], driving a continuous rise in demand for underwater energy. Consequently, efficient management of existing energy resources and development of new energy sources have become research hotspots [2]. The underwater pan-tilt system, as an emerging underwater work platform, primarily carries observation equipment and can adjust the attitude of this equipment within several degrees during underwater engineering operations. However, the pan-tilt system has limited onboard energy storage when operating in deep-sea environments, while task completion is inherently dependent on endurance performance. Furthermore, the complex underwater environment—characterized by factors such as ocean current disturbances, high-pressure corrosion, and long-duration task requirements—poses severe challenges to the pan-tilt system’s endurance capability [3,4]. Traditional underwater pan-tilt systems predominantly employ periodic scanning or constant power consumption modes for energy management strategies. These static strategies exhibit two predominant drawbacks: (1) low temporal synchronization between equipment operation periods and biological occurrence frequency and (2) the inherent trade-off where ensuring monitoring coverage necessitates compromising energy efficiency, consequently reducing device lifespan.

With the proliferation of intelligent technology, underwater pan-tilt technology is currently undergoing transformative innovations. Energy management strategies based on intelligent algorithms are gradually advancing [5]. Among these, reinforcement learning algorithms offer a novel approach to enhancing energy efficiency through real-time environmental perception and decision optimization. Intelligent algorithms now find wide application across various fields. Sufán and Troni et al. [6] introduced a deep reinforcement learning (DRL)-based controller, named the REEF series, for six-degree-of-freedom (6-DOF) underwater vehicles. By integrating thruster usage and signal smoothing penalties into the reward function, the controller learns energy-efficient motion trajectories. While the study demonstrates the capability of DRL to achieve both high-precision control and energy efficiency through real-world underwater experiments, it is also constrained by high computational demands during training and implementation challenges in resource-limited and extreme underwater environments. Rybak L A et al. [7] developed an improved DQN framework for multi-robot collaborative transportation using dueling DQN, which separates the Q-function into state value and advantage components to enhance action assessment and decision-making. However, the approach requires intensive computation during training, substantial sample data, and its adaptability in dynamic environments needs further validation. Lance Chorney [8] developed a multi-agent reinforcement learning (MARL) framework for unmanned underwater vehicle (UUV) dynamic docking control, combining dense/sparse rewards with relative state representation to enable precise local-perception docking. However, RL training complexity and computational costs escalate with task scale, requiring further research for larger-scale UUV fleet control applications. Fang W et al. [9] proposed the IAC-IQL algorithm by integrating Improved Ant Colony Optimization (IAC) with Improved Q-Learning (IQL). This hybrid approach features a multidimensional reward function evaluating path length, smoothness (via Bézier curve constraints), and obstacle avoidance, along with a dynamic reward mechanism to balance exploration and exploitation. While demonstrating excellent static environment performance, the algorithm’s dynamic adaptability in changing environments requires further enhancement to better comply with robotic kinematic constraints in real-world applications. Carlucho, I. et al. [10] developed a DRL-based adaptive control method for AUVs, using three penalty terms to optimize tracking accuracy, energy efficiency, and motion smoothness. The simplified state representation reduces computational load while maintaining effective autonomous control in complex underwater environments, but partial degree-of-freedom control in experiments may constrain the strategy’s generalizability. Liang, Z. et al. [11] proposed a distributed self-organizing cooperative intelligent reconnaissance and combat strategy (CISCS) for multiple UAVs. By designing a finite-time formation controller, a Q-learning improved ant colony algorithm, and an extended artificial potential field method, it achieves rapid formation, efficient task allocation, and internal collision avoidance for UAV swarms in complex environments. However, the study still has limitations including idealized model assumptions, insufficient consideration of dynamic constraints, and lack of in-depth exploration of multi-platform cooperative operations.

Although existing studies have demonstrated significant progress in reinforcement learning/deep reinforcement learning (RL/DRL) for underwater vehicle control tasks such as navigation and docking, critical challenges persist in optimizing energy strategies for pan-tilt systems. Current research primarily focuses on thruster-based multi-degree-of-freedom vehicle motion control, while neglecting the unique energy consumption patterns of pan-tilt mechanisms—rendering existing optimization approaches (e.g., thruster energy penalty or path efficiency improvement) inapplicable. Conventional static control logic shows limitations in dynamic underwater environments (e.g., maintaining stability under ocean current disturbances or achieving adaptive scanning with visibility variations). Moreover, even adaptive RL methods mainly concentrate on large-scale path planning rather than real-time, energy-aware precise adjustments for pan-tilt systems. Most fundamentally, the field lacks systematic investigation into the fundamental trade-offs among algorithmic complexity, monitoring coverage (scanning area/tracking quality), and energy consumption in intelligent pan-tilt control.

Therefore, designing an intelligent optimization strategy that concurrently addresses the specific monitoring objectives (e.g., coverage, target acquisition) and stringent low energy consumption requirements, tailored to the unique dynamics and constraints of underwater pan-tilt scenarios, is crucial for improving underwater equipment operational efficiency and represents a distinct research gap not adequately covered by existing RL approaches for underwater vehicles [12].

This paper addresses the shortcomings of static energy management strategies for underwater pan-tilt systems and the complex, dynamic nature of the underwater environment. By introducing the Q-learning algorithm from the reinforcement learning framework, it provides a new approach to solving the static strategy problem. Compared to algorithms such as CS (Cuckoo Search) and PSO (Particle Swarm Optimization), this algorithm requires no pre-modeling, aligning with the variable characteristics of the underwater environment [13]. By establishing a state–action–reward three-tuple mapping relationship, it continuously interacts with and learns from the environment to obtain optimal decisions. This approach can effectively reduce energy consumption while maintaining high monitoring coverage, thereby significantly extending the operational lifespan of the pan-tilt system.

## 2. Composition and Energy Consumption Modeling of Underwater Pan-Tilt Systems

### 2.1. Underwater Pan-Tilt Systems

**Figure 1:**
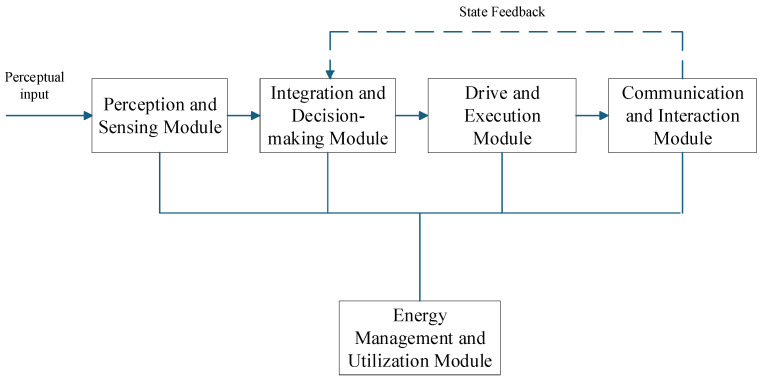
The system composition of the underwater pan-tilt system.

The underwater pan-tilt system, as a typical underwater intelligent operation system, typically employs a “perception-decision-execution” architecture to accomplish complex multi-module assisted operations. The system’s core comprises five modules: the Perception and Sensing Module, the Data Fusion Processing and Decision-Making Module, the Drive and Execution Module, the Communication and Interaction Module, and the Energy Management and Utilization Module (as shown in Figure 1).The Perception and Sensing Module acquires environmental information through visual sensors (cameras) and obtains the pan-tilt unit’s pitch and roll angles via attitude sensors, thereby determining the unit’s own status. The Data Fusion Processing and Decision-Making Module processes sensor-transmitted data in real time using a microcontroller and generates control instructions. The Drive and Execution Module converts control instructions into mechanical movements, driving the pan-tilt unit to perform horizontal rotation and pitch rotation through motors. The Communication and Interaction Module primarily enables the transmission of information obtained by the underwater pan-tilt system to external systems. The Energy Management and Utilization Module optimizes energy usage to ensure prolonged and efficient underwater operation of the pan-tilt system.

### 2.2. Underwater Pan-Tilt Systems’ Energy Consumption Model

The formula for calculating energy consumption of mechanical equipment is as follows:

(1) $$E=\int_{t_{0}}^{t_{1}}P(t)\cdot \frac{1}{\eta (t)}dt$$

where P(t) represents the instantaneous power of the system at time *t* and η(t) represents the instantaneous energy conversion efficiency, with $t_{0}$ and $t_{1}$ indicating the start and end times, respectively.

The overall energy consumption module of the underwater pan-tilt system mainly includes motor, camera, microcontroller unit, and sensor energy consumption. The overall energy consumption model of an underwater pan-tilt system within one working cycle can be expressed as

(2) $$\begin{array}{l} E_{totoal}=\int_{0}^{T}\left[\lambda_{flow(u,\theta )}\cdot P_{motor}+\lambda_{temp(T)}(P_{mcu}+P_{sensor}+P_{camera})\right]dt\\ \lambda_{flow(u,\theta )}=1+k_{u}\left|u(\theta )\right|\\ \lambda_{temp(T)}=1+\alpha_{T}(T-T_{0}) \end{array}$$

where $P_{motor}$ represents the instantaneous power of the motor, $P_{camera}$ denotes the instantaneous power of the camera, $P_{mcu}$ indicates the instantaneous power of the microcontroller unit, $P_{sensor}$ represents the instantaneous power of the sensor, and *T* represents the working cycle of the pan-tilt system. $\lambda_{flow(u,\theta )}$ represents the water flow regulation parameter, which is influenced by the flow velocity and the angle of attack; $k_{u}$ represents the water flow sensitivity coefficient, influenced by the flow velocity; u(θ) denotes the angle-of-attack function; $\lambda_{temp(T)}$ represents the temperature regulation parameter; and $\alpha_{T}$ denotes the temperature drift coefficient, which is device-dependent. T is the reference temperature, typically set to 25 °C, and $T_{0}$ indicates the operating temperature. Notably, the water flow regulation parameter significantly affects motor energy consumption, while other factors are negligible. In contrast, the temperature regulation parameter has a major impact on electronic components.

Under still water (zero flow velocity) and room temperature (25 °C) conditions, perform a time discretization calculation on the above formula:

(3) $$E_{total}=\sum_{k=1}^{N}(P_{motor}[k]+P_{camera}[k]+P_{mcu}[k]+P_{sensor}[k])\Delta t$$

where Δt represents the step size and $N=\frac{T}{\Delta t}$ represents the total number of steps.

The operational mode of underwater pan-tilt systems has undergone a technological evolution from experience-driven approaches to intelligent perception. The traditional timed operation mode relies on marine biologists’ long-term observations of diurnal organism rhythms in target sea areas. It establishes fixed working periods through statistical modeling and uses timers to control the pan-tilt system for scanning observations and video collection within preset intervals. Conversely, the automatic wake-up mode constructs a biological activity probability model by fusing multiple sensor modalities (environmental perception, biological recognition, optical detection, etc.). Upon detecting biological entities, this mode triggers the pan-tilt system’s transition from a dormant to an active state. According to one study in the literature [14], the nocturnal activity frequency of coral reef fish ranges approximately from 7 to 10 times per hour. Considering the variable occurrence frequency of underwater organisms across environments, assume a specific scenario where a target organism appears eight times per hour within the camera’s operational zone. Thus, in automatic wake-up mode, the camera activates eight times hourly, operating for 2 min per activation. In timed mode, the camera operates continuously for 20 min within a designated period. Using Table 1 and Equation (2), the unit time energy consumption for both modes can be derived. The component power consumption and cumulative energy expenditure per unit time for both operational strategies are depicted in the accompanying figure.

**Figure 2:**
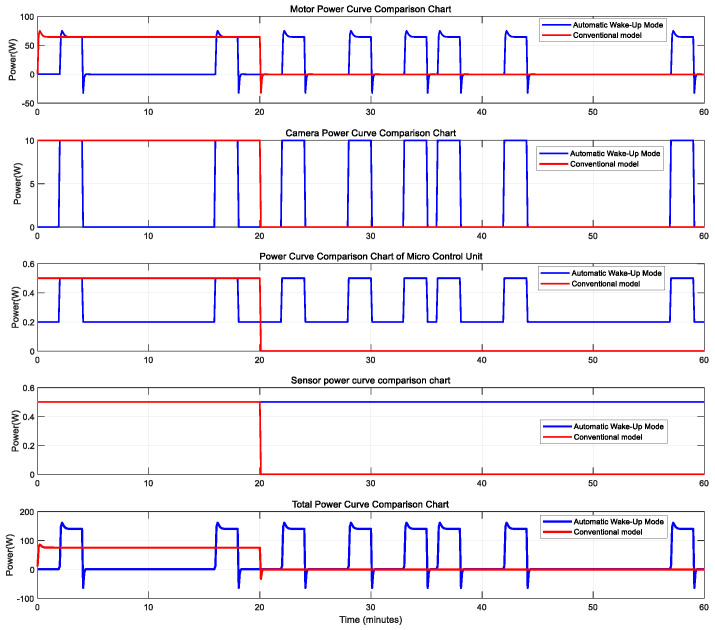
Comparison chart of component power consumption for the two modes.

**Figure 3:**
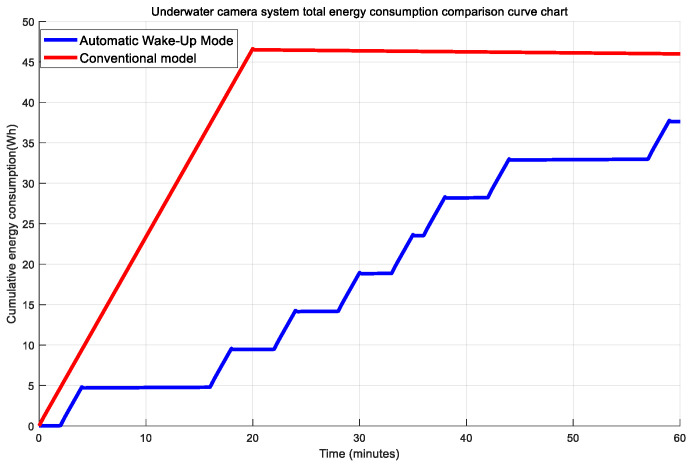
Comparison chart of cumulative energy consumption for the two modes.

Based on the comparative analysis of Figure 2 and Figure 3, the energy efficiency characteristics of the two working modes show significant differences: The traditional timed mode adopts a fixed cycle operation mechanism, and the power consumption of each component shows regular fluctuations. This design has two main drawbacks: Firstly, it maintains a fixed power consumption during periods without biological activity (such as continuous standby of the motor and periodic awakening of the sensor), resulting in ineffective energy consumption. Secondly, due to the sampling interval, it may miss sudden biological behaviors, leading to a decrease in the integrity of the observed data. Compared to traditional timer-based pan-tilt systems, the auto-wakeup mode achieves an energy efficiency improvement of 18.11% per operational cycle under the specified conditions.

(4) $$\gamma =\frac{E_{tradition}-E_{smart}}{E_{tradition}}=\frac{53.67-43.95}{53.67}\approx 18.11\%$$

where γ represents the optimization rate (typically expressed as a percentage), $E_{tradition}$ represents the total energy consumption of the pan-tilt system in conventional mode during the cycle, and $E_{smart}$ indicates the total energy consumption in auto-wake mode during the cycle.

However, the stochasticity of underwater biological activity renders fixed-duration wake-up modes inefficient, as they cannot adapt to dynamic behavioral patterns. To overcome this limitation, we implemented a Q-learning algorithm that dynamically optimizes wake-up strategies through reinforcement learning. This adaptive approach significantly improves both observation efficiency and energy conservation in the system.

## 3. Q-Learning Algorithm for Optimizing Control Strategies

### 3.1. Q-Learning Algorithm

Marine organism activities are influenced by multiple factors including water temperature, illumination, and climatic conditions, while environmental variations at the same location may alter their behavioral rhythms [15,16]. To enhance marine life monitoring while extending pan-tilt systems’ operation duration and conserving energy, implementing rational energy management strategies becomes particularly crucial [17].

The Q-learning algorithm is an unsupervised reinforcement learning algorithm, and it is regarded as one of the commonly used techniques for developing energy management strategies. Through the interaction between the agent and the environment, it aims to understand the value of taking specific actions in a given state. Based on the action values and rewards, it is continuously updated over time to maximize the cumulative rewards [18,19]. The theoretical foundation is built upon the Markov Decision Process (MDP), a fundamental concept in reinforcement learning that provides a principled mathematical framework for modeling the environment as an MDP [20]. A stochastic process is said to possess the Markov property if and only if the state at any given moment depends solely on the state of the previous moment, which can be mathematically expressed as $P(s_{t+1}|s_{t})=P(s_{t+1}|s_{1},\ldots s_{t})$ (where $s_{t}$ represents the current state (at time step *t*); $s_{t+1}$ indicates the resulting next state). In other words, the current state serves as a sufficient statistic for future states—the next state is determined exclusively by the current state and remains unaffected by historical states. The MDP is typically described by the tuple (S, A, P, R, γ), where *S* represents the state space; A denotes the action space; *P* is the state transition probability; R stands for the immediate reward function; γ is the discount factor [21]. In energy management applications, MDPs effectively model system states (e.g., energy storage levels, demand patterns) and controllable actions (e.g., power dispatch, load scheduling) through either discrete formulations suitable for equipment switching scenarios or continuous representations requiring function approximation for precise power allocation. Researchers typically solve these models using value iteration algorithms that recursively apply Bellman equations or policy optimization methods that directly refine control strategies, with solutions often addressing challenges like partial observability through POMDP extensions or multi-agent coordination via decentralized MDP variants. The following Table 2 provides explanations of the parameters related to the MDP decision-making framework for underwater pan-tilt systems.

The key components of Q-learning strictly correspond to the elements in the MDP quintuple. The main key components of the Q-learning algorithm include state space, action selection, reward and punishment functions, and Q-value updating [22,23]. Initialize Q-values first, where the Q-table undergoes continuous updates and refinement through agent–environment interactions. Subsequently, the optimal action is selected and executed for a given state. This action is chosen by the ε−greedy strategy with a probability of 1−ε to select the action with the highest *Q* value at the moment or with a probability of ε to randomly select the preset action. This behavior of choosing a random action can effectively prevent the agent from getting stuck in a local optimal state [24,25]. The formula of the ε−greedy strategy is

(5) $$\pi (s)=\left\{\begin{array}{cc} \arg\max Q(s_{t},a_{t}) & 1-\varepsilon \\ a_{random} & \varepsilon \end{array}\right.$$

where $s_{t}$ represents the state at time *t*, $a_{t}$ denotes the action at time *t*, $\arg\max Q(s_{t},a_{t})$ indicates the action selected when the state and behavior at time *t* obtain a higher Q-value, $a_{random}$ represents a randomly selected action, and ε represents the probability of selecting an action for this strategy. When ε approaches 1, the agent tends to attempt random actions, that is, to explore the environment; when ε approaches 0, the agent tends to utilize the external environment and select the action with the highest action value function [26].

Finally, the agent reacts to the chosen action based on the environment, records the rewards and punishments through the set reward and punishment function, updates the state, and describes the Bellman equation using the time difference method [27]. It continuously updates the Q values according to the specific formula as follows.

(6) $$Q(s,a)\leftarrow Q(s,a)+\alpha [r+\gamma \max_{a\prime }Q(s\prime ,a)-Q(s,a)]$$

where Q(s,a) represents the Q-value obtained after performing action *α* in state *s*; α represents the learning rate, which determines the extent to which new information affects the Q value; *r* indicates the immediate reward obtained after taking the current action; γ represents the discount factor, which measures the weight of future reward; and $\max_{a\prime }Q(s\prime ,a)$ represents the maximum Q value of the best action to be executed in state s′ (which can be understood as the next state).

The Q-learning algorithm, as a classic reinforcement learning algorithm, has its core advantage in the model-free learning approach. It gradually optimizes the strategy through iterative optimization mechanisms and an exploration–exploitation balance mechanism.

### 3.2. Design of Q-Learning Algorithm

Q-learning, as a method of reinforcement learning, is based on the principles of reinforcement learning. By reasonably designing the state space, action space, and reward function, its core mechanism is constructed.

#### 3.2.1. State Space

In order to enable the underwater pan-tilt system to adjust its working mode in a timely manner according to the frequency of underwater organisms’ appearance, rather than simply starting at fixed intervals or frequently, this paper predicts the frequency of organism appearance through the average startup time interval of the camera. It divides the state space *S* into two dimensions, consisting of two discrete variables:

(7) $$s=(s_{i},s_{f})\in S$$

where $s_{i}$ represents the average interval time; $s_{f}$ represents the biological frequency (as a percentage); and *S* denotes the total number of states.

Optimal state space design enhances the agent’s environmental exploration capability, promotes acquisition of high-value control policies, and ultimately improves learning efficiency in underwater sensing systems.

#### 3.2.2. Action Space

In underwater monitoring systems, the requirements for camera observation vary significantly depending on the frequency distribution of different organisms in the environment. To address this variability while maintaining efficient operation, system-level power optimization becomes crucial. This involves dynamic resource adjustment (e.g., adaptive sampling rates) under performance constraints, where “mode partitioning” serves as the core energy-saving strategy to balance ecological observation needs with power efficiency in diverse marine environments [28]. This article divides the automatic wake-up mode of the underwater pan-tilt systems into three types: low-energy mode, standard mode, and high-performance mode, which correspond to three different action spaces.

(8) $$A=(a_{1},a_{2},a_{3})$$

The high-speed response of certain fish species can reach 1500–4000°/s (e.g., the rapid escape behavior of cod), whereas for schooling fish or under low-threat conditions, the response speed may decrease to 200–500°/s. Additionally, these responses are influenced by the surrounding environment. The latency of fish initiation can be as short as 5–10 ms, while in some cases, it may exceed 50 ms [29]. According to the Nyquist theorem, for slow-swimming or stationary fish, a sampling rate greater than 40 Hz is required. A sampling rate of 200 Hz was adopted to account for other influencing factors, incorporating redundancy to ensure optimal system performance. For typical fish activity responses, the sampling rate should exceed 400 Hz; thus, both standard and high-performance modes must operate above 400 Hz with additional redundancy. For high-speed predatory or escape behaviors under threat, an even higher sampling rate is necessary to capture detailed motion information. Considering that the optimal efficiency range for motor operation typically lies between 30% and 90% of the rated speed, operating outside this range leads to reduced efficiency and increased energy consumption. Under constant load conditions, energy consumption rises with increasing motor speed. To prioritize energy savings in low-power mode, the motor operates at 30% of its rated speed, sacrificing some performance. In contrast, standard and high-performance modes require higher responsiveness to track biological activity, necessitating increased motor speeds accordingly.

Accordingly, in low-power mode, the sampling rate is set to 200 Hz (20% of the maximum sampling capability) with 30% of the rated motor speed. Standard mode operates at 650 Hz (65%) paired with 65% motor speed, while high-performance mode employs 850 Hz (85%) and 90% motor speed, aligning with the characteristics of each mode.

The current marine organism appearance frequency is predicted based on historical mean activation intervals of the monitoring system, and the agent selects an action in the action space through the ε−greedy strategy. The action selection procedure of the ε−greedy strategy is implemented as follows [30]:

1. Observe the current state.
2. Generate a random number randomly r∈(0,1).
3. Compare r with ε. If r<ε, the intelligent agent will randomly select an action from the current state’s action set; if r≥ε, the intelligent agent will choose the action with the maximum Q-value in the current state.

In Q-learning algorithms, the design of the action space not only affects the efficiency of Q-value storage and update but also directly influences the exploration strategy, learning progress, and final performance of the agent. An appropriate action space design can help the agent learn more efficiently, avoid overly complex or overly simplistic action selection, and improve the performance and convergence speed of the algorithm.

#### 3.2.3. Reward Function

To further optimize the balance between monitoring accuracy and energy consumption in the pan-tilt system, this study employs a Q-learning algorithm to identify optimal strategies. By predicting the frequency of the appearance of organisms based on the average startup interval time in the early stage, the corresponding working mode is correctly activated and rewards are given. Moreover, the monitoring coefficients and power consumption exhibit significant variations across different operational modes. These factors will jointly affect the calculation of the reward function. To enable the pan-tilt system to make optimal decisions when encountering varying biological occurrence frequencies, we define the reward function *R* for the agent after executing action *α* as follows:

(9) $$\begin{array}{l} R=\lambda \cdot I_{a_{i}=s_{f}}+\eta \cdot M-\mu \cdot E\\ M=\frac{f_{pattern}}{f_{\max}} \end{array}$$

where λ represents the correctness factor of the pattern (experimentally determined as λ = 8); $I_{a_{i}=s_{f}}$ represents the indicator function with subscripts specifying conditions. When the subscript condition is satisfied, it outputs 1; otherwise, it outputs 0. η represents the monitoring factor, μ represents the energy consumption factor, and η and μ are affected by different working modes. *M* indicates the degree of monitoring, and *E* represents the energy consumption value. $f_{pattern}$ represents the actual sampling rate of the current mode. $f_{\max}$ denotes the maximum sampling rate. For computational and experimental convenience, the value is set to 1000.

By reasonably designing the reward function and the reward-punishment factors, the aim is to prevent the reward value from diverging during the training process of the intelligent agent and to accelerate the convergence speed of the intelligent agent [31,32].

### 3.3. Underwater Pan-Tilt Systems’ Energy Management Strategy

Q-learning, as a model-free reinforcement learning algorithm, does not require precise mathematical models of the environment or statistical distributions of biological occurrence frequencies. It can adapt strategies in real time solely through interactive data learning and optimize policies via Formula (6). Compared to methods based on value function, Q-learning has lower computational complexity and can adapt to data-scarce scenarios.

**Figure 4:**
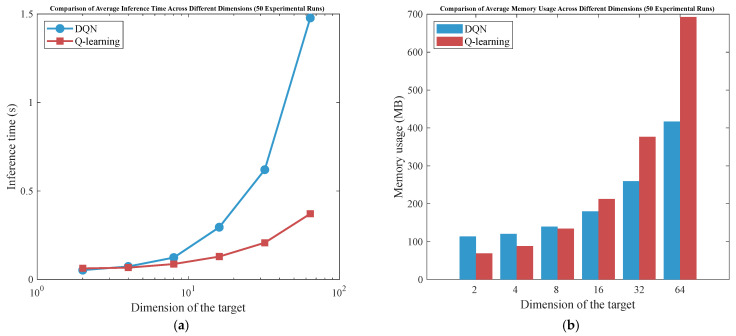
Complexity comparison curves between Q-learning and DQN. (**a**) Comparison of average inference time. (**b**) Comparison of average memory usage.

Although Q-learning requires storing a table of size |*S*| × |*A*| (where S is the state space and A is the action space), its computational complexity per update is O(1) with minimal arithmetic operations (a single Bellman update). In contrast, value function approximation (e.g., DQN) relies on deep neural networks. The training process of deep neural networks faces several key computational challenges: (1) it incurs high computational overhead during both forward and backward propagation phases, (2) requires significant memory capacity to store network parameters, and (3) exhibits strong hardware dependencies, particularly relying on GPU acceleration to achieve practical training speeds. These constraints collectively pose substantial barriers to efficient model development and deployment. For our specific problem, the state space is deliberately designed to be compact: S is discretized into N_s_ × N_f_ states (N_s_ = 3 time-interval bins, N_f_ = 3 frequency bins, |S| = 9),|A|= 3(low/standard/high-performance modes). Thus, the Q-table has only 9 × 3 = 27 entries, making storage and updates trivial for embedded systems. This design choice prioritizes deployability in resource-limited underwater pan-tilt system where real-time inference must run on microcontroller. Value approximation would incur unnecessary complexity without tangible benefits for such small-scale problems (as shown in Figure 4).

Compared with Q-learning, DQN exhibits significantly higher inference time and memory consumption in low-dimensional scenarios. Particularly for embedded systems or resource-constrained devices, the complexity of DQN introduces additional computational and storage overhead. Therefore, Q-learning represents a more suitable choice in such cases, especially when dealing with small state and action spaces.

For underwater pan-tilt system working environments, where the frequency of marine life appearances is influenced by diurnal cycles, seasonal variations, and migratory behaviors, Q-learning proves particularly suitable. Moreover, integrating Q-learning into the underwater pan-tilt system’s automatic wake-up mode—compared to fixed single-observation durations—enables dynamic selection of operational modes (with varying durations) based on different biological occurrence frequencies. This approach ensures sufficient monitoring coverage while conserving energy and extending operational time.

This study presents an intelligent energy management strategy for underwater pan-tilt observation systems to address the limitations of fixed observation modes and enhance adaptability to varying marine biological activity frequencies. The proposed solution implements a three-tier adaptive wake-up mechanism (low-power, standard, and high-performance modes) that dynamically adjusts system operation based on real-time biological activity patterns while maintaining required monitoring performance. Initial operation in standard mode establishes baseline biological activity through calculated average activation intervals, enabling predictive mode selection. During low-frequency biological activity periods, the system prioritizes energy conservation by activating low-power mode with minimized monitoring intensity. Conversely, high-frequency biological activity triggers high-performance mode with optimized energy consumption and maximized monitoring capability. Through continuous reinforcement learning optimization, the system progressively refines its decision-making algorithm to achieve an optimal trade-off between monitoring effectiveness (quantified by monitoring factor) and energy efficiency (measured by energy consumption factor), ultimately achieving extended operational duration while maintaining reliable observation performance in dynamic underwater environments. The specific steps are as follows:

1. Set the learning rate α and exploration rate γ for the intelligent agent.
2. Initialization of the agent function.
3. Set the parameters for the state space, action space and reward function.
4. The agent acquires the current state and makes action selections using the ε-greedy strategy.
5. Update the Q-value continuously through the reward function.
6. Repeat steps 4 and 5, and iterate until the Q value stabilizes.

Although the Q-learning algorithm may cause large energy consumption fluctuations in the initial exploration stage due to random strategies, adjusting the ε-greedy strategy parameters reasonably is essential for more comprehensive environmental exploration and avoiding local optima.

**Figure 5:**
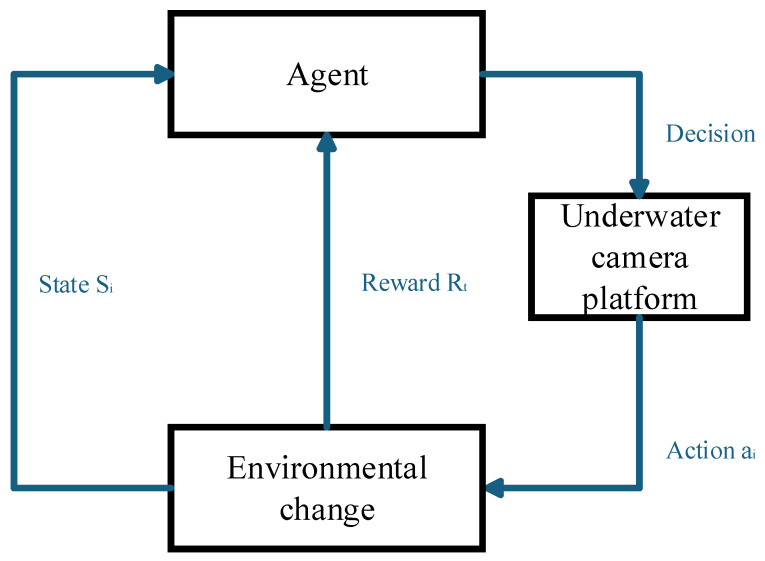
Flowchart of underwater cloud platform Q-learning algorithm.

The energy management strategy of underwater pan-tilt systems is optimized using Q-learning algorithm to derive an optimal energy consumption scheme. A detailed flowchart is provided to demonstrate the core implementation steps and operational workflow, as shown in Figure 5.

## 4. Simulation Experiments and Result Analysis

### 4.1. Parameter Settings

This paper selects the average time interval and the frequency of biological occurrence as the state inputs and divides the discrete intervals according to the actual requirements. $s_{i}$ can be divided into (0,2],(2,5],(5,+∞), and $s_{f}$ can be divided into (0,5],(5,20],(20,+∞). For the action outputs, discrete intervals are also divided according to actual needs. At the same time, relevant parameters for executing different actions are set, as shown in Table 3.

Notably, when no marine organisms are detected within the observation range of the pan-tilt system (i.e., in non-operational state), the system switches to standby mode with a power consumption of 0.18 Wh/min. While increasing the dimensionality of action and state sets improves control precision, it simultaneously leads to higher Q-table dimensionality and consequently slows algorithm convergence. Therefore, parameter design must carefully balance control accuracy against computational efficiency [33].

To enable the underwater pan-tilt agent to achieve optimal task-specific performance while enhancing decision-making capability and learning efficiency, proper configuration of Bellman equation parameters is crucial. The learning rate requires careful consideration: an excessively large value may lead to algorithmic instability, while an overly small value could result in unacceptably slow convergence. Similarly, the discount factor determines the trade-off between immediate and future rewards. A value approaching 1 emphasizes long-term rewards, whereas a value near 0 prioritizes immediate gains.

**Figure 6:**
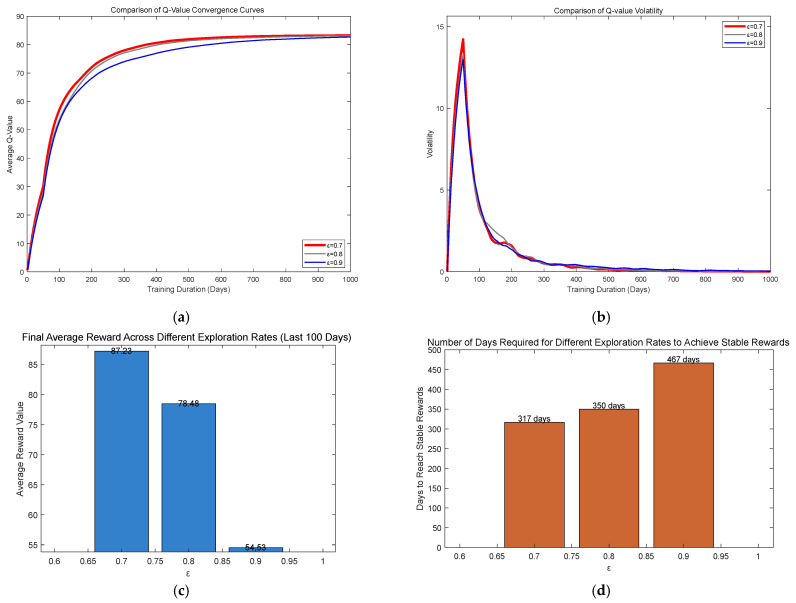
Comparison of different exploration rates. (**a**) Comparison of Q-value convergence curves. (**b**) Q-value oscillation analysis across varying exploration rates. (**c**) Final average reward across different exploration rates. (**d**) Days to reach stable rewards.

The exploration rate reflects the trade-off between exploration and exploitation during the agent’s learning process. Given the initial environmental uncertainty, the agent tends to prioritize exploration, hence a higher initial exploration rate (typically 0.7–0.9). As environmental familiarity increases, the agent gradually shifts toward exploiting known experiences to optimize its policy, resulting in a lower exploration rate. In this study, considering requirements such as low energy consumption, high monitoring efficiency, easy deployment, and low cost—along with convergence speed and fluctuation stability—we compared three initial exploration rates with relatively high values (Figure 6). The results demonstrate that ε = 0.7 outperforms the other two options in terms of convergence speed, policy stability, and final reward attainment, thus best meeting the design specifications.

Therefore, systematic optimization of the learning rate and discount factor as well as reasonable selection of the exploration rate are essential for balanced performance [34], as shown in Table 4.

### 4.2. Experimental Analysis and Results

To maintain the natural randomness of marine organism appearances, the emergence frequency in simulation experiments follows an exponential distribution. This stochastic modeling approach ensures that the underwater pan-tilt system’s activation frequency realistically corresponds to biological occurrence patterns, as follows:

(10) $$f(\lambda ,t)=\lambda e^{-\lambda t}$$

**Figure 7:**
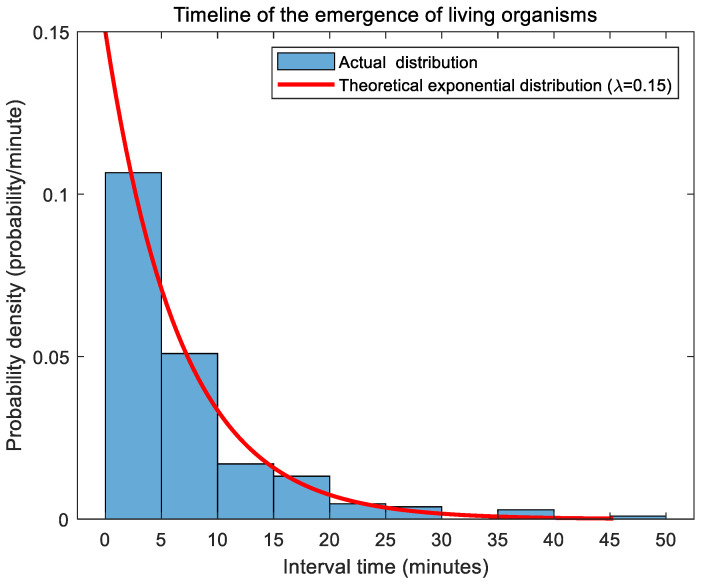
Exponential distribution plot of underwater organism appearance intervals.

Set λ=0.15. Its histogram is shown in Figure 7.

**Figure 8:**
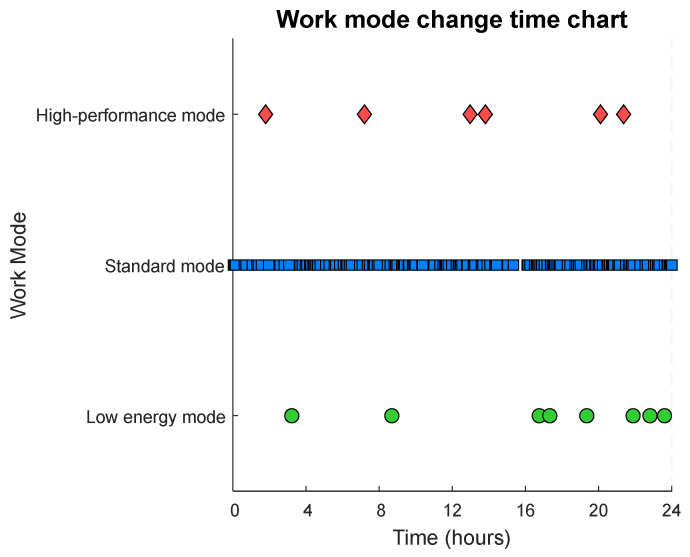
Twenty-four-hour diagram of the camera pan-tilt mode changes.

Based on the Q-learning algorithm, the energy consumption of the underwater pan-tilt system facing different frequencies of biological appearances is optimized. To enable the pan-tilt system to predict the frequency of biological appearances in the current observation environment as accurately as possible, its decision-making logic is designed accordingly. In the initial stage, the pan-tilt system operates in standard mode for the first four cycles, recording and calculating the average activation interval time over these four cycles. This average activation interval time is then compared with a predefined threshold range to determine the appropriate operational mode for the pan-tilt system. Through MATLAB (version: R2024b) simulations, it is demonstrated that the underwater pan-tilt system, optimized using the Q-learning algorithm, can adapt its working mode based on the frequency of biological appearances. By predicting the frequency of biological occurrences via the average activation interval time, the system activates the corresponding operational state, as shown in Figure 8.

Since the predictive decisions are made based on the average interval time of the first four occurrences of the standard mode, the situation where the standard mode is dominant is more common.

**Figure 9:**
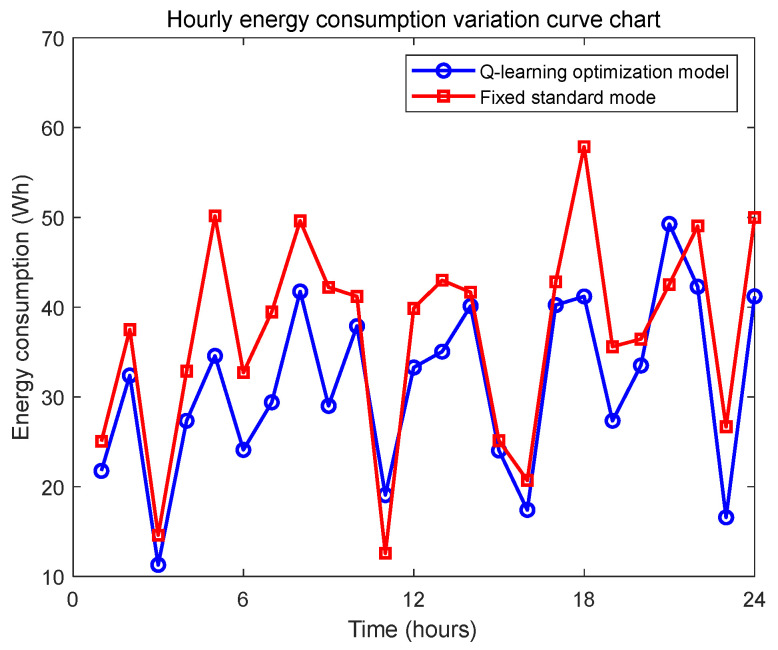
Hourly energy consumption change comparison chart.

**Figure 10:**
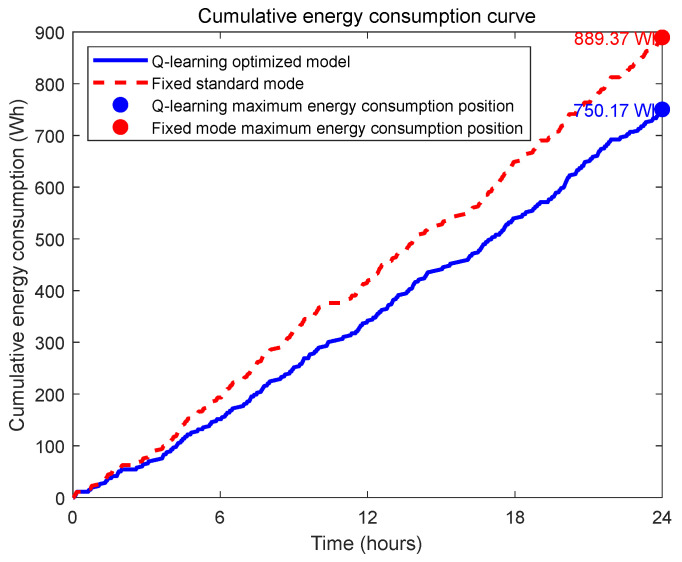
Twenty-four-hour cumulative energy consumption comparison chart.

To systematically validate the effectiveness of the Q-learning algorithm in optimizing the dynamic energy consumption of the underwater pan-tilt system, this study designed a comparative experiment. The experimental group employs an intelligent control strategy based on reinforcement learning, dynamically adjusting the pan-tilt system’s operational modes (e.g., low-power mode, standard mode, high-performance mode) by predicting state parameters such as biological appearance frequency. In contrast, the control group adopts a fixed standard operational mode, where the unit remains in standard observation mode whenever biological activity is detected. The 24 h real-time energy consumption curves (Figure 9, individual seed results) are used to compare the energy consumption fluctuation characteristics between the two groups, while cumulative energy consumption trend graphs (Figure 10, individual seed results) are plotted to evaluate energy-saving efficiency.

Based on the analysis of the aforementioned energy consumption comparison charts, although the fixed standard mode occasionally exhibits slightly lower instantaneous energy consumption than the Q-learning optimized solution at certain moments, the overall trend demonstrates that the underwater pan-tilt system dynamically regulated by the Q-learning algorithm significantly outperforms the fixed standard mode in terms of energy efficiency per unit time. This advantage becomes even more evident in the cumulative energy consumption graph, where the energy-saving effect progressively amplifies over time. The key differences lie in the Q-learning solution’s ability to intelligently switch operational modes, effectively avoiding unnecessary energy waste, while automatically reducing power consumption during non-critical monitoring periods. This adaptive approach ultimately achieves the goal of energy conservation.

**Figure 11:**
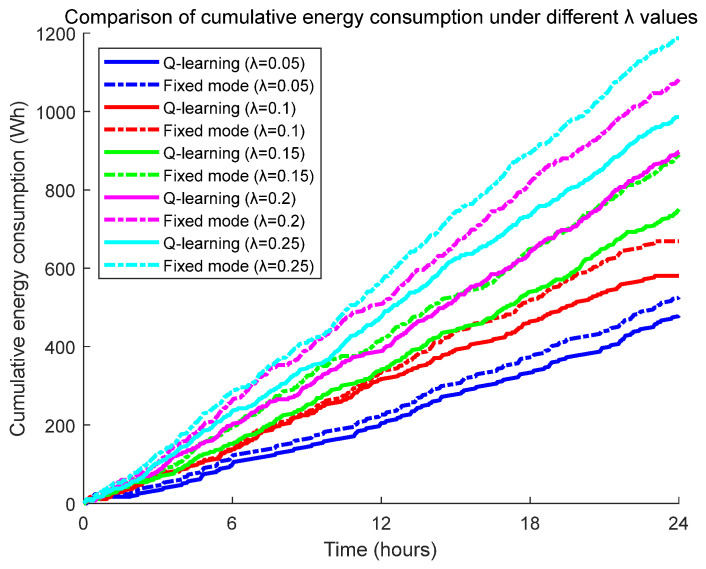
Cumulative energy consumption comparison chart for different λ values.

**Figure 12:**
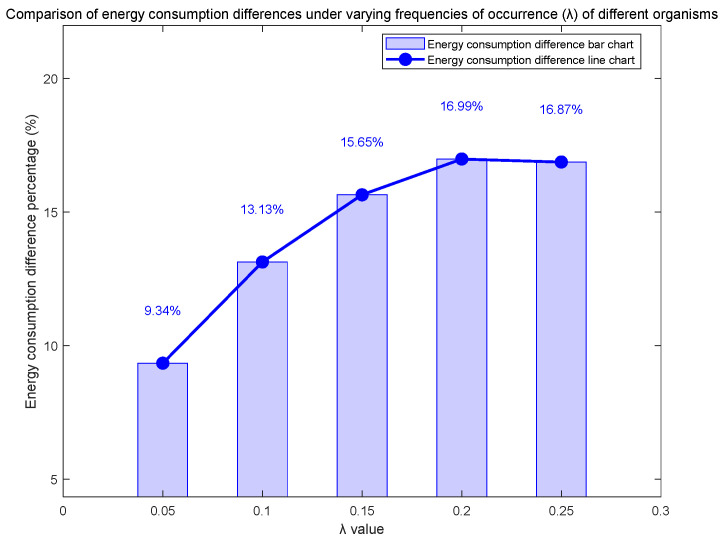
Comparison chart of energy consumption differences under different λ values.

To further investigate the energy optimization performance of the Q-learning-based dynamic control strategy under varying biological appearance frequencies, this study employs exponential distributions with different parameters to simulate gradient changes in biological occurrence rates. Through a 24 h continuous monitoring experiment, we systematically analyze the cumulative energy consumption trends of the pan-tilt system, with a focus on examining the dynamic differences in energy consumption between the two operational modes caused by variations in biological appearance frequencies, as illustrated in Figure 11 and Figure 12.

The experimental data analysis demonstrates that under different parameter values (biological occurrence frequencies), the pan-tilt system employing the Q-learning dynamic optimization strategy consistently exhibits superior energy efficiency compared to the fixed-duration observation mode. Notably, as the biological occurrence frequency increases within a reasonable range, the energy-saving advantage of the Q-learning algorithm shows a significant upward trend. This indicates that the intelligent control strategy exhibits enhanced adaptability for high-frequency biological monitoring scenarios.

**Figure 13:**
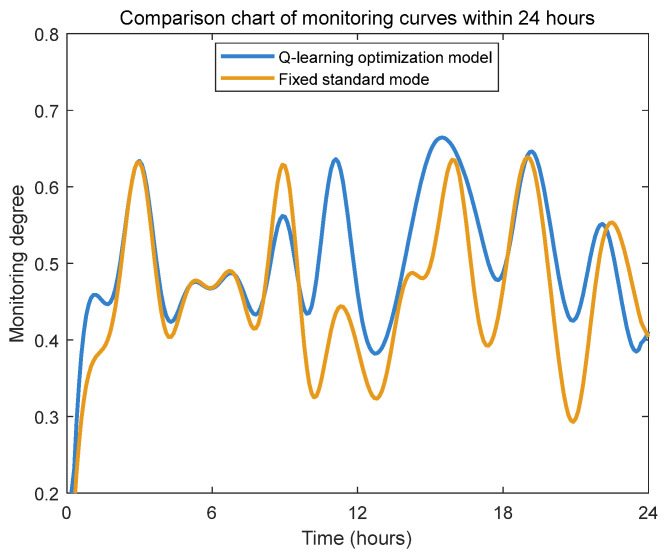
Twenty-four-hour monitoring curve change comparison chart.

**Figure 14:**
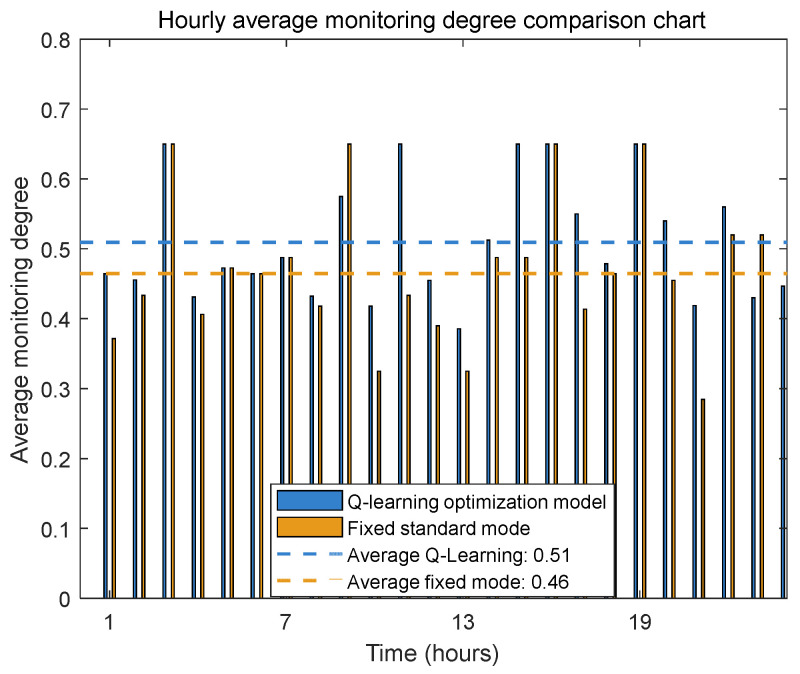
Hourly average monitoring degree comparison chart.

To validate whether the Q-learning algorithm can maintain essential monitoring performance while optimizing energy consumption of the underwater pan-tilt system, this study designed a comparative experimental scheme. First, differentiated monitoring intensity parameters and monitoring factors were configured for different working modes based on Table 3. Subsequently, through comparative testing between the Q-learning dynamic optimization mode and the fixed standard mode, we systematically evaluated the performance differences in monitoring metrics when λ = 0.15 (as shown in Figure 13 and Figure 14).

Comparative analysis of the monitoring performance between the two modes (as illustrated in Figure 13 and Figure 14) reveals that the Q-learning dynamic optimization mode achieves marginally superior overall monitoring quality compared to the fixed standard mode, with an approximate five-percentage-point improvement in average monitoring intensity.

**Figure 15:**
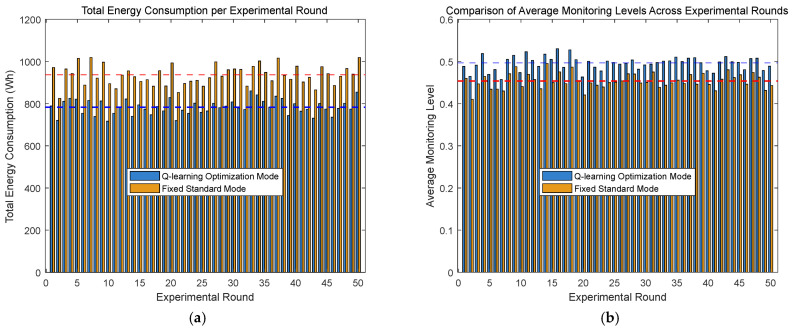
Comparative analysis of energy consumption and monitoring performance across multiple experimental rounds. (**a**) Energy consumption comparison. (**b**) Monitoring performance comparison.

To ensure the stability and reliability of experimental results, as the outcomes from a single experimental seed may be affected by random factors, multiple rounds of seeds (50 rounds of seeds are used in this study) were introduced for validation while keeping other experimental conditions unchanged. The results are shown in the following Figure 15.

As shown in the figure above, multiple rounds of experimental data demonstrate that the Q-learning optimization mode still outperforms the fixed standard mode in terms of total energy consumption and monitoring accuracy. The specific numerical results are presented in Table 5 below.

**Figure 16:**
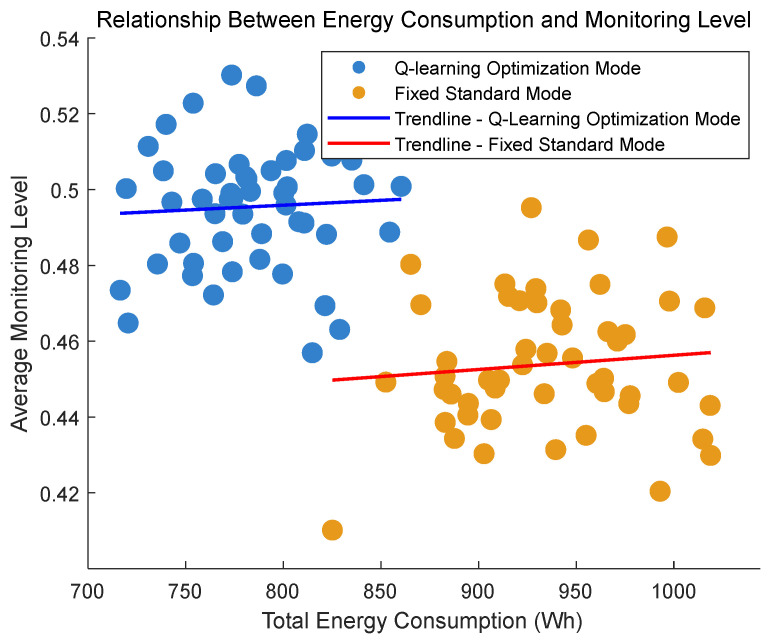
Scatter plot of energy consumption vs. monitoring accuracy.

**Figure 17:**
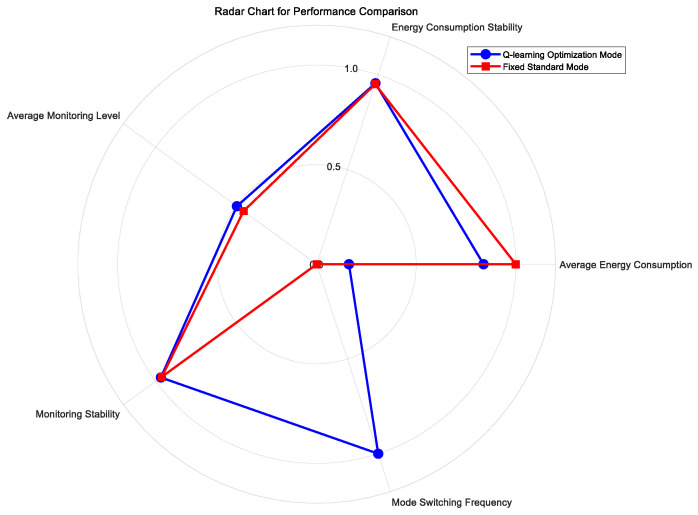
Radar chart of performance comparison.

**Figure 18:**
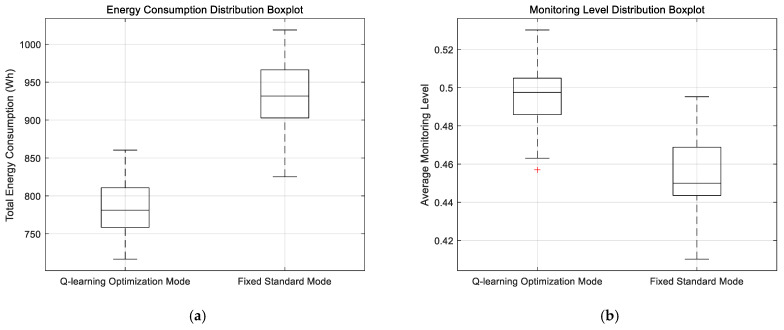
Box plot of energy consumption vs. monitoring accuracy. (**a**) Comparative box plot of energy consumption. (**b**) Comparative box plot of monitoring degree.

To further investigate the stability and distribution patterns of energy consumption and monitoring accuracy under both modes, scatter plots, box plots, and radar charts were introduced. As shown in Figure 16, Figure 17 and Figure 18.

The analysis of the presented figures shows that both monitoring modes achieve slightly better performance with higher energy consumption. However, the Q-learning-optimized mode outperforms the fixed standard mode in overall performance. Under the same monitoring level, the Q-learning mode consumes significantly less energy. This advantage comes from its intelligent algorithm that dynamically optimizes resource allocation to reduce energy waste. Meanwhile, it maintains high monitoring accuracy without sacrificing coverage. The radar chart clearly illustrates the Q-learning mode’s advantages across key metrics. Besides energy efficiency and monitoring accuracy, it also shows good stability with balanced performance in all indicators. Both modes maintain stable trends in their energy–performance curves. This stability confirms that the Q-learning optimization provides reliable controllability and predictability without causing significant performance fluctuations.

In summary, the Q-learning-based dynamic optimization scheme demonstrates superior performance in underwater gimbal operations compared to fixed modes. By dynamically adjusting to monitoring demands, it achieves sustained energy savings (with cumulative efficiency gains) while improving average monitoring accuracy by 11.11%. This dual optimization stems from the algorithm’s adaptive responsiveness, which strategically reduces non-essential energy expenditure without compromising precision during critical operational phases. The results validate Q-learning’s practical efficacy in resource-constrained marine environments.

## 5. Conclusions

The complexity of underwater environments imposes heightened demands on energy resources. Research into energy optimization strategies for underwater pan-tilt systems reveals a technological evolution from passive scheduling to intelligent decision-making. Traditional time-triggered operation modes rely on prior statistical models of biological activity patterns, executing full-power scans at fixed intervals, resulting in significant idle energy consumption. In contrast, an intelligent wake-up mode employs a multi-modal feature recognition network to transition the pan-tilt system from a dormant state to an active state. When combined with adaptive observation techniques, this approach achieves markedly lower energy consumption per operational cycle compared to conventional methods. To ensure efficient underwater operation, this study comparatively analyzes the per-cycle energy consumption of a pan-tilt system operating under traditional time-triggered modes versus those equipped with an intelligent auto-wake-up model. Furthermore, we innovatively integrate a Q-learning algorithm into the auto-wake-up framework to further optimize energy savings and enhance monitoring efficiency. The conclusions are as follows: (1) Compared with traditional timer-based pan-tilt systems, the auto-wakeup-enabled underwater pan-tilt unit demonstrates significant energy efficiency improvements under specified conditions, achieving an 18.11% reduction in power consumption per operational cycle. (2) To address the stochastic nature of underwater biological occurrences, we implemented a Q-learning algorithm to dynamically optimize the operational states of the underwater pan-tilt unit according to varying frequencies of biological presence. Compared to the fixed-duration monitoring in conventional auto-wake-up mode, our approach achieves a 16.21% reduction in energy consumption while simultaneously improving monitoring coverage by 11.11%. Although this study has achieved preliminary results in intelligent energy optimization for underwater pan-tilt systems, certain limitations remain. Notably, the combined effects of environmental factors such as water current velocity and light intensity variations were not considered, which may affect both energy efficiency optimization and monitoring accuracy. Future research should focus on incorporating additional sensor data and real-time feedback mechanisms to enhance system robustness and intelligent performance.

## Figures and Tables

**Table 1:** Theoretical power consumption of underwater pan-tilt system equipment.

Parameter	Numerical/W
The working power consumption of the motor	75
The standby power consumption of the motor	1.5
The power consumption of the camera during operation	10
The standby power consumption of the camera	1
Power consumption during the wake-up process of the microcontroller unit	0.5
Power consumption during the sleep mode of the microcontroller unit	0.02
Power consumption of underwater sensors on the underwater pan-tilt-system	0.5
Power consumption of the photoelectric sensor	0.5

**Table 2:** Transformation of underwater pan-tilt system energy consumption model.

Key Components in MDP Decision-Making Framework	Energy Consumption Model of Underwater Pan-Tilt System	Design Elements
Environment	Energy Consumption System	Simulation Environment
Action	Real-time Rules	Mode Transition Logic
State	Runtime Status	Operational Mode Characteristics
Reward	Reward Mechanism	Key Performance Indicators

**Table 3:** Motion space-related parameters.

Action Space	Working Hours (Min)	Energy Consumption (Wh)	Monitoring Degree	η	μ
Low energy mode	1	1.5	0.2	0.15	0.7
Standard mode	3	5.5	0.65	0.55	0.55
High-performance mode	5	9	0.85	0.8	0.25

**Table 4:** Parameters related to Q-learning agents.

Parameter	Numerical
α	0.2
β	0.95
ε	0.7

**Table 5:** Mean and standard deviation of parameters from multiple experimental runs.

Mode	Mean Total Energy Consumption (Wh)	Total Energy Std Dev (Wh)	Mean Monitoring Accuracy	Monitoring Accuracy Std Dev
Q-learning	783.52	±35.15	0.50	±0.02
Fixed Standard Mode	935.05	±46.59	0.45	±0.02

## Data Availability

The original contributions presented in this study are included in the article material. Further inquiries can be directed to the corresponding author.
